# Supplementary material for: SW16-7, a Novel Ackermannviridae Bacteriophage with Highly Effective Lytic Activity Targets Salmonella enterica Serovar Weltevreden
Source: Microorganisms. 2023 Aug 15;11(8):2090. doi: 10.3390/microorganisms11082090 (PMC10458263; doi:10.3390/microorganisms11082090)
Supplement: Supplementary file 1 [file microorganisms-11-02090-s001.zip › Supplement/Table S1 General features and database matches of predicted proteins encoded by Salmonella phage SW16-7.docx]

Table S1 General features of predicted CDSs encoded by Salmonella phage SW16-7

| CDS | Product | Length | Direction | Position (from) | Postion(to) | Gene Function |
| --- | --- | --- | --- | --- | --- | --- |
| CDS1 | hypothetical protein | 234 | forward | 3 | 236 | Hypothetical protein |
| CDS2 | hypothetical protein | 375 | reverse | 263 | 637 | Hypothetical protein |
| CDS3 | putative uncharacterised protein | 606 | reverse | 634 | 1239 | Hypothetical protein |
| CDS4 | Phage protein | 309 | reverse | 1232 | 1540 | Phage morphogenesis |
| CDS5 | hypothetical protein | 207 | reverse | 1636 | 1842 | Hypothetical protein |
| CDS6 | hypothetical protein | 573 | reverse | 1891 | 2463 | Hypothetical protein |
| CDS7 | DNA polymerase | 3000 | forward | 2543 | 5542 | DNA metabolism |
| CDS8 | hypothetical protein | 342 | forward | 5605 | 5946 | Hypothetical protein |
| CDS9 | hypothetical protein | 777 | forward | 5943 | 6719 | Hypothetical protein |
| CDS10 | hypothetical protein | 306 | forward | 6729 | 7034 | Hypothetical protein |
| CDS11 | P-loop kinase-2 | 906 | forward | 7034 | 7939 | DNA metabolism |
| CDS12 | hypothetical membrane protein | 198 | forward | 7942 | 8139 | Hypothetical protein |
| CDS13 | hypothetical protein | 381 | forward | 8136 | 8516 | Hypothetical protein |
| CDS14 | hypothetical membrane protein | 186 | forward | 8497 | 8682 | Hypothetical protein |
| CDS15 | aGPT-Pplase2 | 1233 | forward | 8679 | 9911 | DNA metabolism |
| CDS16 | hypothetical membrane protein | 168 | forward | 9948 | 10115 | Hypothetical protein |
| CDS17 | hypothetical protein | 213 | forward | 10198 | 10410 | Hypothetical protein |
| CDS18 | hypothetical protein | 387 | forward | 10407 | 10793 | Hypothetical protein |
| CDS19 | hypothetical protein | 1116 | forward | 10790 | 11905 | Hypothetical protein |
| CDS20 | hypothetical protein | 570 | forward | 11924 | 12493 | Hypothetical protein |
| CDS21 | hypothetical protein | 129 | forward | 12498 | 12626 | Hypothetical protein |
| CDS22 | hypothetical protein | 423 | forward | 12626 | 13048 | Hypothetical protein |
| CDS23 | hypothetical protein | 366 | forward | 13111 | 13476 | Hypothetical protein |
| CDS24 | hypothetical protein | 447 | forward | 13476 | 13922 | Hypothetical protein |
| CDS25 | hypothetical protein | 204 | forward | 13919 | 14122 | Hypothetical protein |
| CDS26 | hypothetical protein | 129 | forward | 14139 | 14267 | Hypothetical protein |
| CDS27 | hypothetical protein | 348 | forward | 14278 | 14625 | Hypothetical protein |
| CDS28 | RIIA protein | 2757 | forward | 14732 | 17488 | DNA metabolism |
| CDS29 | RIIB protein | 1566 | forward | 17520 | 19085 | DNA metabolism |
| CDS30 | [Salmonella phage Vi01] | 162 | reverse | 19251 | 19412 | Phage morphogenesis |
| CDS31 | hypothetical protein | 426 | forward | 19411 | 19836 | Hypothetical protein |
| CDS32 | hypothetical protein | 393 | forward | 19866 | 20258 | Hypothetical protein |
| CDS33 | Phage tail fiber | 810 | forward | 20237 | 21046 | Phage morphogenesis |
| CDS34 | hypothetical protein | 231 | forward | 21049 | 21279 | Hypothetical protein |
| CDS35 | putative histone-like protein | 513 | forward | 21368 | 21880 | DNA metabolism |
| CDS36 | hypothetical protein | 198 | forward | 21926 | 22123 | Hypothetical protein |
| CDS37 | hypothetical protein | 489 | forward | 22166 | 22654 | Hypothetical protein |
| CDS38 | putative uncharacterised protein | 585 | forward | 22651 | 23235 | Hypothetical protein |
| CDS39 | DNA topoisomerase | 1914 | forward | 23285 | 25198 | DNA metabolism |
| CDS40 | DNA topoisomerase | 1332 | forward | 25200 | 26531 | DNA metabolism |
| CDS41 | hypothetical membrane protein | 288 | forward | 26574 | 26861 | Hypothetical protein |
| CDS42 | hypothetical protein | 312 | forward | 26864 | 27175 | Hypothetical protein |
| CDS43 | Arn.3 conserved hypothetical protein | 411 | forward | 27179 | 27589 | Hypothetical protein |
| CDS44 | hypothetical protein | 243 | forward | 27640 | 27882 | Hypothetical protein |
| CDS45 | Tk.4 protein | 600 | forward | 27879 | 28478 | Auxiliary metabolism |
| CDS46 | DexA exonuclease | 633 | forward | 28479 | 29111 | Auxiliary metabolism |
| CDS47 | hypothetical protein | 306 | forward | 29108 | 29413 | Hypothetical protein |
| CDS48 | hypothetical protein | 213 | forward | 29477 | 29689 | Hypothetical protein |
| CDS49 | putative uncharacterised protein | 687 | forward | 29686 | 30372 | Hypothetical protein |
| CDS50 | hypothetical protein | 342 | forward | 30372 | 30713 | Hypothetical protein |
| CDS51 | putative serine/threonine protein phosphatase | 561 | forward | 30713 | 31273 | Auxiliary metabolism |
| CDS52 | hypothetical protein | 414 | forward | 31308 | 31721 | Hypothetical protein |
| CDS53 | hypothetical protein | 339 | forward | 31718 | 32056 | Hypothetical protein |
| CDS54 | hypothetical protein | 342 | forward | 32127 | 32468 | Hypothetical protein |
| CDS55 | Cd allosteric enzyme | 507 | forward | 32468 | 32974 | Auxiliary metabolism |
| CDS56 | hypothetical membrane protein | 408 | forward | 32985 | 33392 | Hypothetical protein |
| CDS57 | Phage head completion protein | 615 | reverse | 33604 | 34218 | Phage morphogenesis |
| CDS58 | Phage baseplate protein (T4-like gp48) | 969 | forward | 34269 | 35237 | Phage morphogenesis |
| CDS59 | Phage baseplate wedge subunit | 555 | forward | 35249 | 35803 | Phage morphogenesis |
| CDS60 | hypothetical protein | 1389 | forward | 35800 | 37188 | Hypothetical protein |
| CDS61 | hypothetical protein | 1947 | forward | 37200 | 39146 | Hypothetical protein |
| CDS62 | Phage DNA helicase loader | 666 | reverse | 39147 | 39812 | DNA metabolism |
| CDS63 | putative homing endonuclease | 696 | reverse | 39805 | 40500 | DNA metabolism |
| CDS64 | putative uncharacterised protein | 240 | reverse | 40497 | 40736 | Hypothetical protein |
| CDS65 | hypothetical membrane protein | 258 | reverse | 40714 | 40971 | Hypothetical protein |
| CDS66 | hypothetical protein | 216 | reverse | 40976 | 41191 | Hypothetical protein |
| CDS67 | DNA ligase | 1425 | reverse | 41191 | 42615 | DNA metabolism |
| CDS68 | hypothetical protein | 342 | reverse | 42729 | 43070 | Hypothetical protein |
| CDS69 | hypothetical protein | 351 | reverse | 43125 | 43475 | Hypothetical protein |
| CDS70 | hypothetical protein | 213 | reverse | 43477 | 43689 | Hypothetical protein |
| CDS71 | hypothetical membrane protein | 117 | reverse | 43689 | 43805 | Hypothetical protein |
| CDS72 | hypothetical protein | 1194 | reverse | 43802 | 44995 | Hypothetical protein |
| CDS73 | hypothetical protein | 321 | reverse | 45124 | 45444 | Hypothetical protein |
| CDS74 | hypothetical protein | 336 | reverse | 45460 | 45795 | Hypothetical protein |
| CDS75 | DNA primase/helicase | 1428 | reverse | 45858 | 47285 | DNA metabolism |
| CDS76 | hypothetical protein | 330 | reverse | 47292 | 47621 | Hypothetical protein |
| CDS77 | UvsX RecA-like recombination protein | 1086 | reverse | 47599 | 48684 | DNA metabolism |
| CDS78 | hypothetical protein | 543 | reverse | 48669 | 49211 | Hypothetical protein |
| CDS79 | putative dUTP diphosphatase | 555 | reverse | 49211 | 49765 | Auxiliary metabolism |
| CDS80 | hypothetical protein | 570 | reverse | 49762 | 50331 | Hypothetical protein |
| CDS81 | putative thymidylate synthase | 1047 | reverse | 50328 | 51374 | DNA metabolism |
| CDS82 | P-loop kinase-1 | 663 | reverse | 51374 | 52036 | DNA metabolism |
| CDS83 | hypothetical protein | 867 | reverse | 52111 | 52977 | Hypothetical protein |
| CDS84 | hypothetical protein | 288 | reverse | 53154 | 53441 | Hypothetical protein |
| CDS85 | hypothetical protein | 750 | reverse | 53462 | 54211 | Hypothetical protein |
| CDS86 | Gp2 DNA end protector protein | 705 | reverse | 54275 | 54979 | Phage morphogenesis |
| CDS87 | Phage baseplate hub protein (T4-like gp54) | 945 | forward | 55033 | 55977 | Phage morphogenesis |
| CDS88 | Single stranded DNA-binding protein, phage-associated | 1047 | reverse | 56004 | 57050 | DNA metabolism |
| CDS89 | hypothetical protein | 240 | reverse | 57149 | 57388 | Hypothetical protein |
| CDS90 | Gp33 T4-like late promoter transcription accessory protein | 246 | reverse | 57396 | 57641 | Gene expression |
| CDS91 | Regulatory protein, FmdB family | 246 | reverse | 57634 | 57879 | Gene expression |
| CDS92 | hypothetical membrane protein | 312 | reverse | 57866 | 58177 | Hypothetical protein |
| CDS93 | hypothetical protein | 591 | reverse | 58177 | 58767 | Hypothetical protein |
| CDS94 | hypothetical protein | 525 | reverse | 59288 | 59812 | Hypothetical protein |
| CDS95 | Phage baseplate hub subunit (T4-like gp26) | 807 | forward | 59862 | 60668 | Phage morphogenesis |
| CDS96 | hypothetical protein | 150 | forward | 60668 | 60817 | Hypothetical protein |
| CDS97 | Phage baseplate hub protein (T4-like gp5) | 1608 | forward | 61179 | 62786 | Phage morphogenesis |
| CDS98 | Phage baseplate wedge subunit (T4-like gp25) | 381 | forward | 62862 | 63242 | Phage morphogenesis |
| CDS99 | hypothetical protein | 351 | reverse | 63239 | 63589 | Hypothetical protein |
| CDS100 | hypothetical protein | 462 | reverse | 63595 | 64056 | Hypothetical protein |
| CDS101 | putative glutaredoxin | 225 | reverse | 64162 | 64386 | Auxiliary metabolism |
| CDS102 | Ribonucleotide reductase of class Ia (aerobic), beta subunit (EC 1.17.4.1) | 1104 | reverse | 64396 | 65499 | Auxiliary metabolism |
| CDS103 | Ribonucleotide reductase of class Ia (aerobic), alpha subunit (EC 1.17.4.1) | 2283 | reverse | 65572 | 67854 | Auxiliary metabolism |
| CDS104 | Phosphate starvation-inducible protein PhoH, predicted ATPase | 843 | reverse | 67938 | 68780 | Auxiliary metabolism |
| CDS105 | Phage endolysin | 795 | reverse | 68885 | 69679 | Lysis |
| CDS106 | putative uncharacterised protein | 489 | reverse | 69879 | 70367 | Hypothetical protein |
| CDS107 | hypothetical protein | 207 | reverse | 70563 | 70769 | Hypothetical protein |
| CDS108 | Phage-associated DNA primase | 1059 | reverse | 70766 | 71824 | DNA metabolism |
| CDS109 | hypothetical protein | 612 | reverse | 71824 | 72435 | Hypothetical protein |
| CDS110 | MobD.6 conserved hypothetical phage protein | 354 | reverse | 72493 | 72846 | Hypothetical protein |
| CDS111 | hypothetical protein | 240 | reverse | 72897 | 73136 | Hypothetical protein |
| CDS112 | hypothetical protein | 570 | reverse | 73146 | 73715 | Hypothetical protein |
| CDS113 | phage associated protein | 2550 | reverse | 73789 | 76338 | Phage morphogenesis |
| CDS114 | hypothetical protein | 360 | reverse | 76420 | 76779 | Hypothetical protein |
| CDS115 | hypothetical protein | 624 | reverse | 76900 | 77523 | Hypothetical protein |
| CDS116 | conserved uncharacterised protein | 615 | reverse | 77523 | 78137 | Hypothetical protein |
| CDS117 | hypothetical protein | 279 | reverse | 78237 | 78515 | Hypothetical protein |
| CDS118 | NrdA.1 conserved hypothetical protein | 330 | reverse | 78496 | 78825 | Hypothetical protein |
| CDS119 | hypothetical protein | 309 | reverse | 78822 | 79130 | Hypothetical protein |
| CDS120 | Phage recombination-related endonuclease Gp46 CDS | 2337 | reverse | 79133 | 81469 | DNA metabolism |
| CDS121 | Cell division trigger factor (EC 5.2.1.8) | 1116 | reverse | 81471 | 82586 | Auxiliary metabolism |
| CDS122 | Gp55 T4-like sigma factor involved in late transcription | 729 | reverse | 82586 | 83314 | Gene expression |
| CDS123 | Ribonuclease HI (EC 3.1.26.4) | 528 | reverse | 83326 | 83853 | DNA metabolism |
| CDS124 | hypothetical protein | 768 | forward | 83899 | 84666 | Hypothetical protein |
| CDS125 | DNA helicase | 1719 | reverse | 84663 | 86381 | DNA metabolism |
| CDS126 | DNA-binding protein HU-beta | 279 | reverse | 86510 | 86788 | DNA metabolism |
| CDS127 | hypothetical protein | 285 | reverse | 86879 | 87163 | Hypothetical protein |
| CDS128 | hypothetical protein | 663 | reverse | 87165 | 87827 | Hypothetical protein |
| CDS129 | hypothetical protein | 900 | reverse | 87885 | 88784 | Hypothetical protein |
| CDS130 | hypothetical protein | 315 | reverse | 88836 | 89150 | Hypothetical protein |
| CDS131 | hypothetical protein | 687 | reverse | 89150 | 89836 | Hypothetical protein |
| CDS132 | hypothetical protein | 450 | reverse | 89916 | 90365 | Hypothetical protein |
| CDS133 | hypothetical protein | 288 | reverse | 90487 | 90774 | Hypothetical protein |
| CDS134 | hypothetical protein | 375 | reverse | 90771 | 91145 | Hypothetical protein |
| CDS135 | hypothetical protein | 240 | reverse | 91205 | 91444 | Hypothetical protein |
| CDS136 | hypothetical protein | 324 | reverse | 91923 | 92246 | Hypothetical protein |
| CDS137 | hypothetical protein | 2202 | reverse | 92289 | 94490 | Hypothetical protein |
| CDS138 | hypothetical protein | 171 | reverse | 94483 | 94653 | Hypothetical protein |
| CDS139 | Nicotinamide phosphoribosyltransferase | 1665 | reverse | 94695 | 96359 | Auxiliary metabolism |
| CDS140 | Ribose-phosphate pyrophosphokinase | 855 | reverse | 96356 | 97210 | Auxiliary metabolism |
| CDS141 | hypothetical protein | 795 | reverse | 97321 | 98115 | Hypothetical protein |
| CDS142 | hypothetical protein | 402 | reverse | 98096 | 98497 | Hypothetical protein |
| CDS143 | hypothetical protein | 372 | reverse | 98558 | 98929 | Hypothetical protein |
| CDS144 | hypothetical protein | 2019 | reverse | 98973 | 100991 | Hypothetical protein |
| CDS145 | aGPT-Pplase1 | 1221 | reverse | 101106 | 102326 | DNA metabolism |
| CDS146 | hypothetical protein | 870 | reverse | 102396 | 103265 | Hypothetical protein |
| CDS147 | Phage endoribonulcease translational repressor of early genes, regA | 465 | reverse | 103282 | 103746 | Gene expression |
| CDS148 | Phage DNA polymerase clamp loader subunit Gp62 | 423 | reverse | 103776 | 104198 | DNA metabolism |
| CDS149 | Replication factor C small subunit / Phage DNA polymerase clamp loader subunit | 990 | reverse | 104203 | 105192 | DNA metabolism |
| CDS150 | hypothetical protein | 657 | reverse | 105192 | 105848 | Hypothetical protein |
| CDS151 | Sliding clamp DNA polymerase accessory protein, phage associated | 669 | reverse | 105930 | 106598 | DNA metabolism |
| CDS152 | hypothetical protein | 378 | forward | 106939 | 107316 | Hypothetical protein |
| CDS153 | RNA-DNA and DNA-DNA helicase UvsW | 1509 | reverse | 107307 | 108815 | DNA metabolism |
| CDS154 | hypothetical protein | 573 | reverse | 108851 | 109423 | Hypothetical protein |
| CDS155 | hypothetical protein | 747 | reverse | 109439 | 110185 | Hypothetical protein |
| CDS156 | putative DNA repair/recombination protein UvsY | 456 | reverse | 110185 | 110640 | DNA metabolism |
| CDS157 | Phage tail completion protein | 501 | reverse | 110683 | 111183 | Phage morphogenesis |
| CDS158 | hypothetical protein | 651 | forward | 111213 | 111863 | Hypothetical protein |
| CDS159 | hypothetical protein | 729 | reverse | 111865 | 112593 | Hypothetical protein |
| CDS160 | hypothetical protein | 168 | reverse | 112632 | 112799 | Hypothetical protein |
| CDS161 | hypothetical protein | 423 | reverse | 112841 | 113263 | Hypothetical protein |
| CDS162 | hypothetical protein | 321 | reverse | 113269 | 113589 | Hypothetical protein |
| CDS163 | hypothetical protein | 282 | reverse | 113649 | 113930 | Hypothetical protein |
| CDS164 | hypothetical protein | 237 | reverse | 114035 | 114271 | Hypothetical protein |
| CDS165 | hypothetical protein | 441 | reverse | 114280 | 114720 | Hypothetical protein |
| CDS166 | hypothetical protein | 225 | reverse | 114738 | 114962 | Hypothetical protein |
| CDS167 | hypothetical protein | 201 | reverse | 115023 | 115223 | Hypothetical protein |
| CDS168 | hypothetical protein | 333 | reverse | 115283 | 115615 | Hypothetical protein |
| CDS169 | hypothetical protein | 852 | reverse | 115700 | 116551 | Hypothetical protein |
| CDS170 | Phage major capsid protein of Caudovirales (T4-like gp23) | 1323 | reverse | 116655 | 117977 | Phage morphogenesis |
| CDS171 | Phage prohead assembly (scaffolding) protein (T4-like gp22) | 864 | reverse | 118069 | 118932 | Phage morphogenesis |
| CDS172 | Phage prohead assembly (scaffolding) protein (T4-like gp21) | 666 | reverse | 118978 | 119643 | Phage morphogenesis |
| CDS173 | hypothetical protein | 306 | reverse | 119654 | 119959 | Hypothetical protein |
| CDS174 | hypothetical protein | 168 | reverse | 119970 | 120137 | Hypothetical protein |
| CDS175 | Phage portal (connector) protein (T4-like gp20) | 1692 | reverse | 120175 | 121866 | Phage morphogenesis |
| CDS176 | Phage tail tube protein | 534 | reverse | 121933 | 122466 | Phage morphogenesis |
| CDS177 | hypothetical protein | 795 | forward | 122554 | 123348 | Hypothetical protein |
| CDS178 | Phage tail sheath protein (T4-like gp18) | 1896 | reverse | 123374 | 125269 | Phage morphogenesis |
| CDS179 | Phage terminase, large subunit (T4-like gp17) | 2211 | reverse | 125322 | 127532 | Phage morphogenesis |
| CDS180 | Phage terminase, small subunit (T4-like gp16) | 702 | reverse | 127513 | 128214 | Phage morphogenesis |
| CDS181 | Phage tail completion protein (T4-like gp15) | 696 | reverse | 128217 | 128912 | Phage morphogenesis |
| CDS182 | Phage head completion protein | 651 | reverse | 128915 | 129565 | Phage morphogenesis |
| CDS183 | hypothetical protein | 213 | forward | 129625 | 129837 | Hypothetical protein |
| CDS184 | Unclassified head-tail protein | 753 | reverse | 129865 | 130617 | Phage morphogenesis |
| CDS185 | hypothetical protein | 339 | reverse | 130607 | 130945 | Hypothetical protein |
| CDS186 | hypothetical protein | 249 | reverse | 130929 | 131177 | Hypothetical protein |
| CDS187 | Phage virulence-associated VriC protein | 4839 | reverse | 131229 | 136067 | holin and lysis genes |
| CDS188 | Phage tailspike | 2238 | reverse | 136161 | 138398 | Phage morphogenesis |
| CDS189 | Phage tailspike | 3603 | reverse | 138646 | 142248 | Phage morphogenesis |
| CDS190 | hypothetical protein | 1212 | reverse | 142300 | 143511 | Hypothetical protein |
| CDS191 | hypothetical protein | 855 | reverse | 143514 | 144368 | Hypothetical protein |
| CDS192 | Phage baseplate wedge subunit (T4-like gp6) | 1782 | reverse | 144349 | 146130 | Phage morphogenesis |
| CDS193 | hypothetical protein | 588 | forward | 146481 | 147068 | Hypothetical protein |
| CDS194 | hypothetical protein | 147 | forward | 147111 | 147257 | Hypothetical protein |
| CDS195 | tRNA-Met-CAT | 74 | forward | 149624 | 149697 | tRNA genes |
| CDS196 | tRNA-Arg-TCT | 74 | forward | 149777 | 149850 | tRNA genes |
| CDS197 | recognition of host receptor | 1335 | forward | 149998 | 151332 | Phage morphogenesis |
| CDS198 | tRNA-Asn-GTT | 72 | forward | 151462 | 151533 | tRNA genes |
| CDS199 | tRNA-Ser-GCT | 86 | forward | 152101 | 152186 | tRNA genes |
| CDS200 | tRNA-Pro-CGG | 86 | forward | 152193 | 152278 | tRNA genes |
| CDS201 | phage associated protein CDS | 237 | forward | 152465 | 152701 | Phage morphogenesis |
| CDS202 | conserved phage associated protein | 519 | forward | 152710 | 153228 | Phage morphogenesis |
| CDS203 | hypothetical protein | 663 | forward | 153299 | 153961 | Hypothetical protein |
| CDS204 | hypothetical protein | 1266 | forward | 154031 | 155296 | Hypothetical protein |
| CDS205 | hypothetical protein | 222 | forward | 155443 | 155664 | Hypothetical protein |
